# Supplementary material for: Intestinal fungi are causally implicated in microbiome assembly and immune development in mice
Source: Nat Commun. 2020 May 22;11:2577. doi: 10.1038/s41467-020-16431-1 (PMC7244730; doi:10.1038/s41467-020-16431-1)
Supplement: Supplementary file 1 — Supplementary Information [file 41467_2020_16431_MOESM1_ESM.pdf]

Supplementary Information

Intestinal fungi are causally implicated in microbiome assembly and immune development in mice.

van Tilburg Bernardes *et al.* (2020)

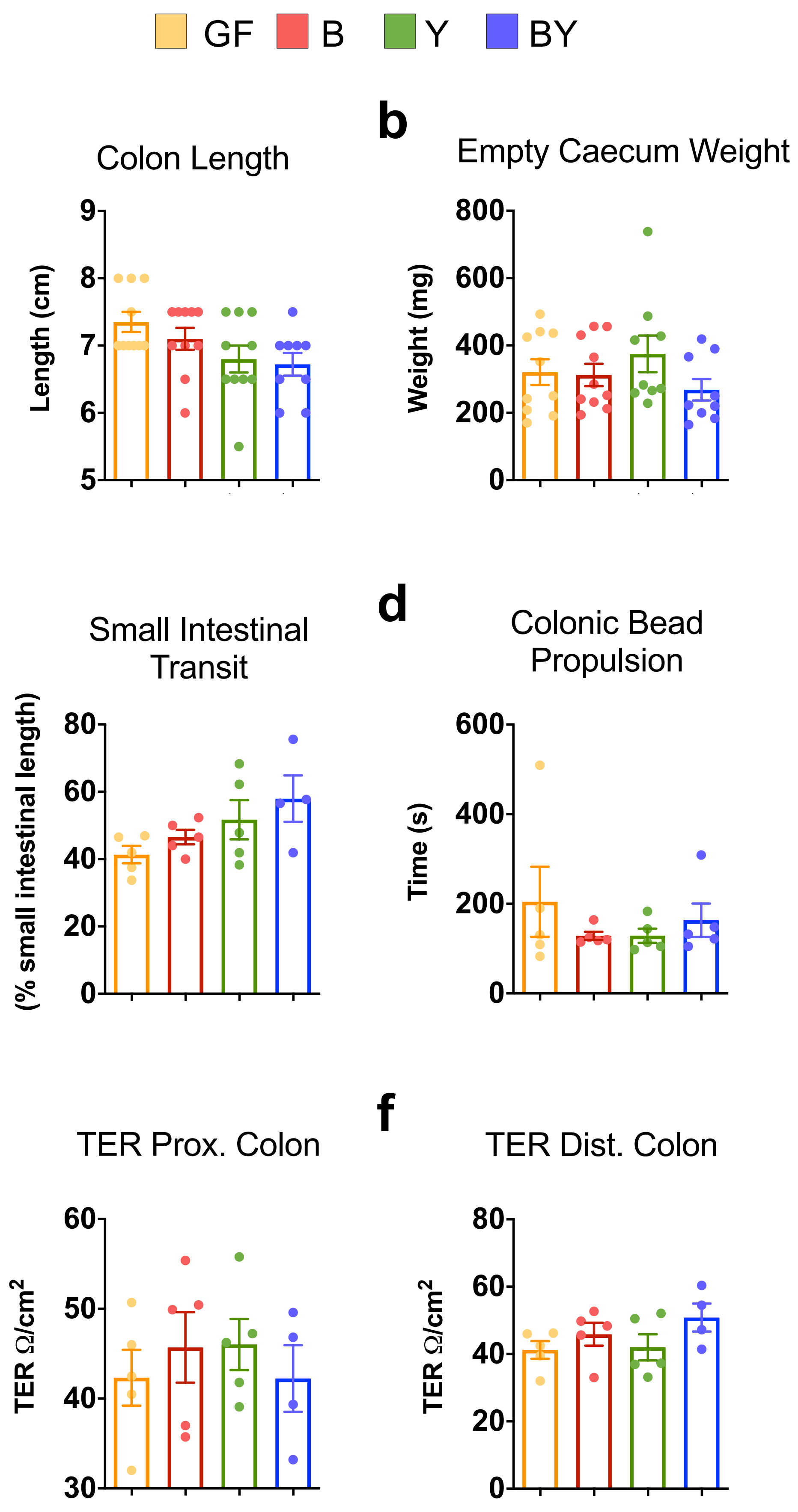

Supplementary Fig. 1: Microbial colonization does not impact intestinal transit and colonic functionality. (a) Large intestine length and **(b)** empty caecum weight of gnotobiotic dams. **(c)** Small intestine transit determined by movement of non-absorbable dye. **(d)** Large intestine transit determined by colonic bead propulsion. **(e)** Proximal and **(f)** distal colon paracellular permeability measured by electrical Transepithelial Resistance (TER). (a-f) Data expressed as mean ± S.E.M.; color denotes colonization treatment (GF = yellow, B = red, Y = green, BY = royal blue); (a) N_{GF}=10, N_B=10, N_Y=10, N_{BY}=9; (b) N_{GF}=10, N_B=10, N_Y=9, N_{BY}=9; (c, e-f) N_{GF}=5, N_B=5, N_Y=5, N_{BY}=4; (d) N_{GF}=5, N_B=5, N_Y=5, N_{BY}=5; no statistically significant differences between groups (one-way ANOVA and Tukey posthoc tests). Source data are provided as a Source Data file.

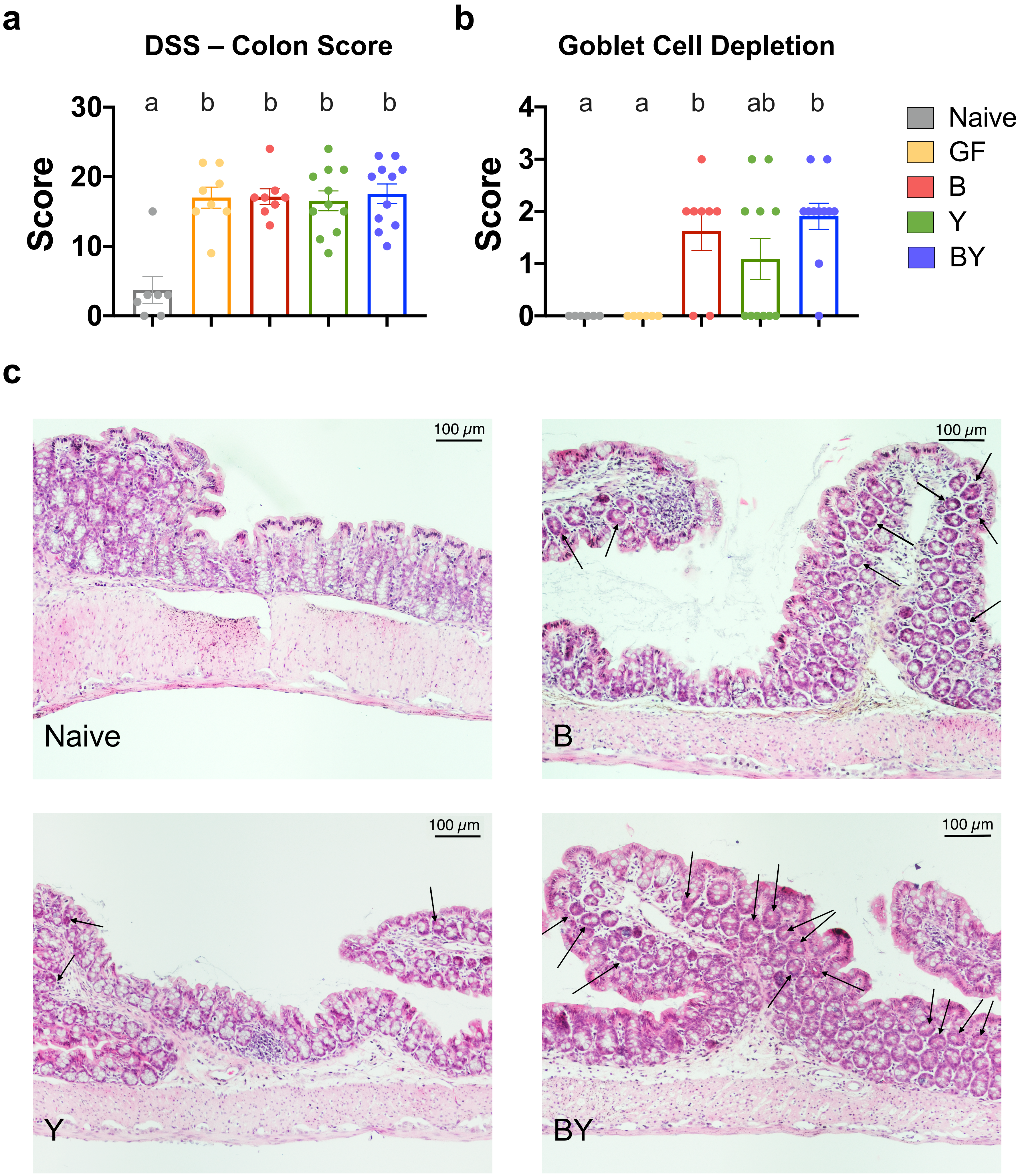

Supplementary Fig. 2: DSS-induced colitis in gnotobiotic mice. (a) Total histopathological inflammation score for DSS-challenged colon. Inflammation score was assessed by sum of pathological measurements, including total inflammation, crypt damage, crypt abscess and goblet cell depletion. **(b)** Results for goblet cell depletion. Presence (1) or absence (0) of goblet cell depletion was multiplied by degree score focal (1), patchy (2), or diffuse (3). (a-b) Data expressed as mean ± S.E.M.; color denotes colonization and DSS treatment (Naive = gray, GF = yellow, B = red, Y = green, BY = royal blue); N_{NAIVE}=7, N_{GF}=8, N_{B}=8, N_{Y}=11, N_{BY}=11; different letters above bars indicate statistically significant differences defined by one-way ANOVA and Tukey posthoc tests; $P<0.05$. **(c)** Representative hematoxylin and eosin (H&E)-stained sections from one gnotobiotic experiment. Arrows indicate regions of goblet cell depletion. Scale bar, 100 µm. Source data are provided as a Source Data file.

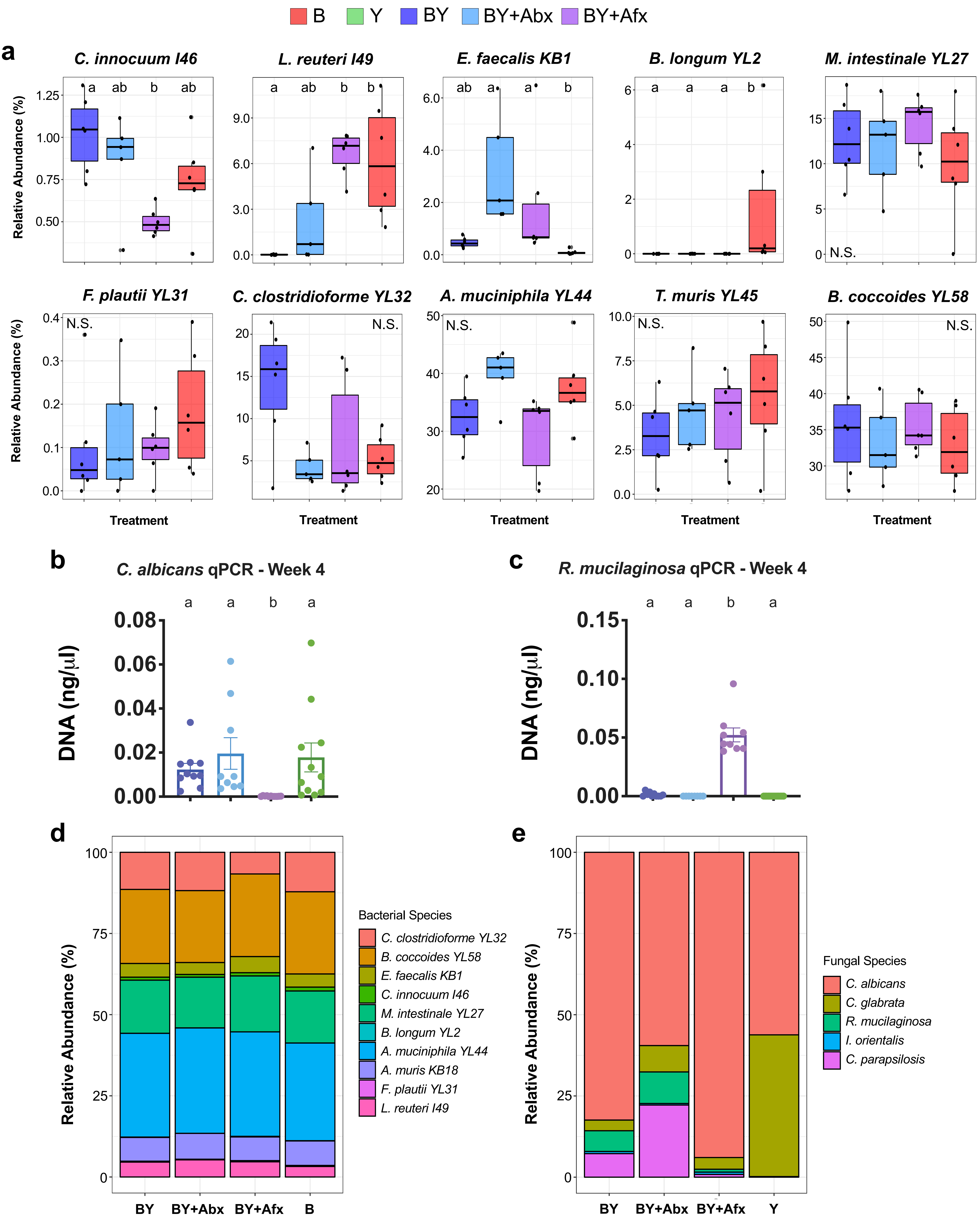

Supplementary Fig. 3

Supplementary Fig. 3: Fungal colonization and antimicrobial treatments impact microbial community composition. Ecological community analyses of 16S and ITS2 sequences. **(a)** Relative abundances of bacterial species at 4 weeks. qPCR quantification (standard curve method) of **(b)** *C. albicans* and **(c)** *R. mucilaginosa* DNA in fecal samples. Relative abundance plots of **(d)** bacterial and **(e)** fungal species at 9 weeks. (a-c) Color denotes colonization treatment (B = red, Y = green, BY = royal blue, BY+Abx = cyan blue, BY+Afx = purple); (d-e) Color denotes microbial species as indicated by graphs legend; (a) Boxplots indicate median (inside line) and 25% and 75% percentiles as the lower and upper hinges, respectively; N_B=6, N_Y=8, N_{BY}=6, N_{Abx}=5, N_{Afx}=6; (b-c) data combined from two different experiments and expressed as mean ± S.E.M.; N_Y=14, N_{BY}=12, N_{Abx}=10, N_{Afx}=12; (d-e) Coloured bozes in abundance plots denote median percentage abundance per taxa; N_B=10, N_Y=11, N_{BY}=10, N_{Abx}=7, N_{Afx}=12. Different letters above bars indicate statistically significant differences defined by (a, d) Kruskal-Wallis with post-hoc Dunn tests and FDR corrected or (b-c) one-way ANOVA and Tukey posthoc tests; $P<0.05$; N.S. no significant differences. Source data are provided as a Source Data file.

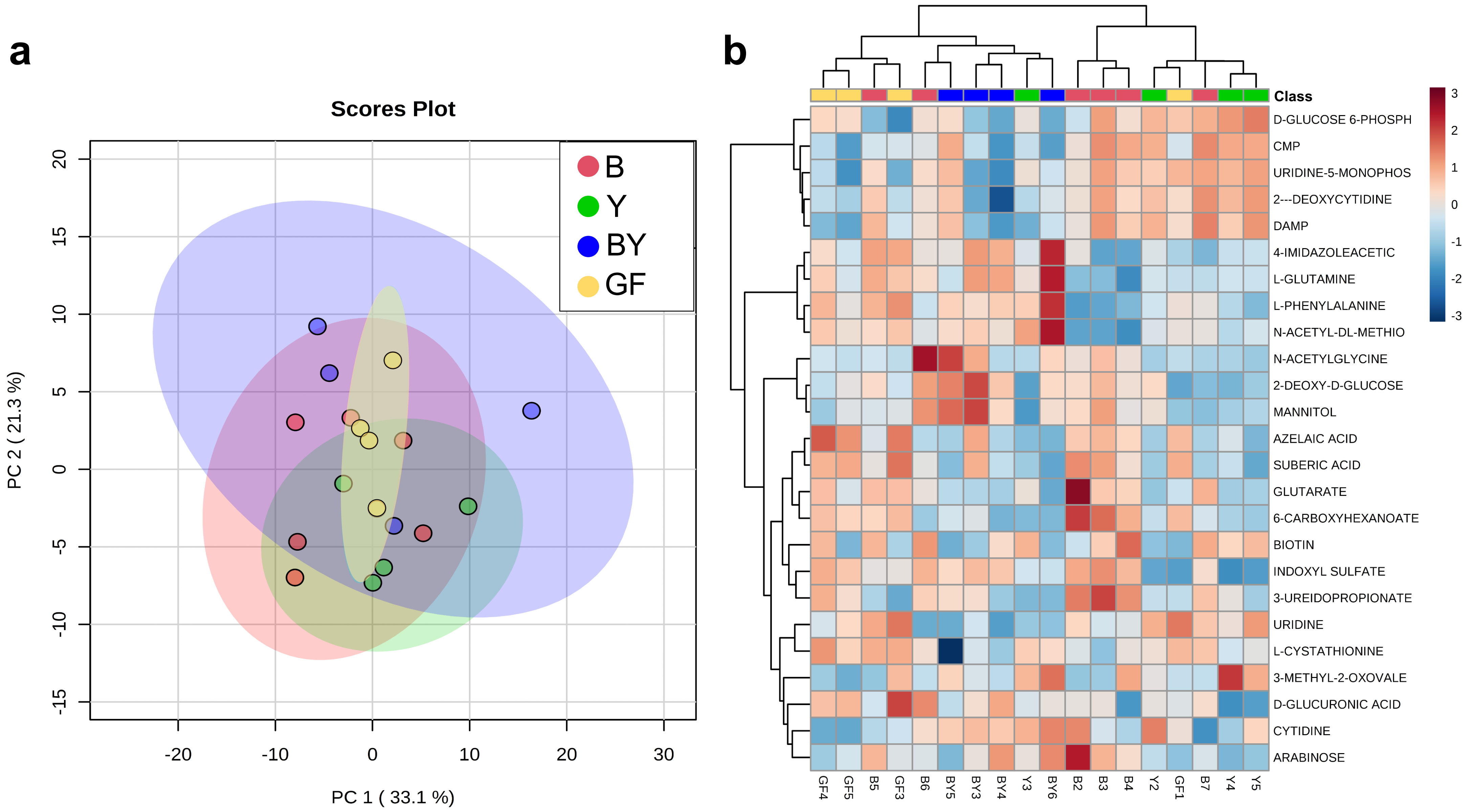

Supplementary Fig. 4: Metabolic profiles of small intestine content is not different among gnotobiotic groups. (a) Principal component analysis score plot of 124 metabolites detected in small bowel fecal content of gnotobiotic mice groups at 4 weeks of age. **(b)** Heat map of expression of top 25 metabolites detected in small bowel contents at 4 weeks. (a-b) Color denotes colonization treatment (GF = yellow, B = red, Y = green, BY = royal blue); N_{GF}=4, N_B=6, N_Y=4, N_{BY}=4; no statistically significant differences between groups (one-way ANOVA with Fisher's post-hoc FDR correction). Source data are provided as a Source Data file.

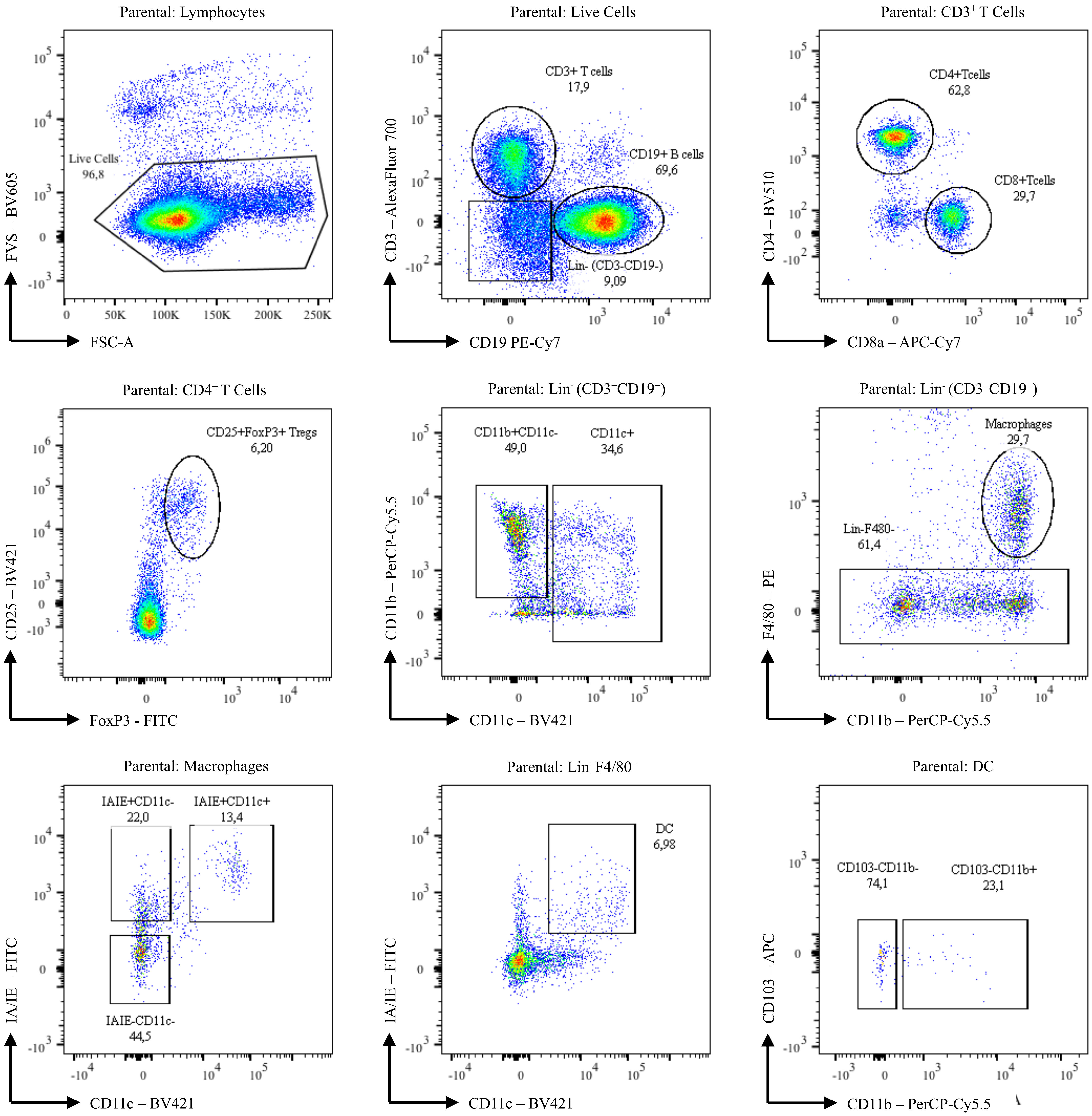

Supplementary Fig. 5: Flow cytometry gating strategy. Spleen immune cells were stained with intra- and extracellular marker-specific antibodies and sorted by flow cytometry (see methods). Analysis performed in FlowJo™ version 10.5.3.

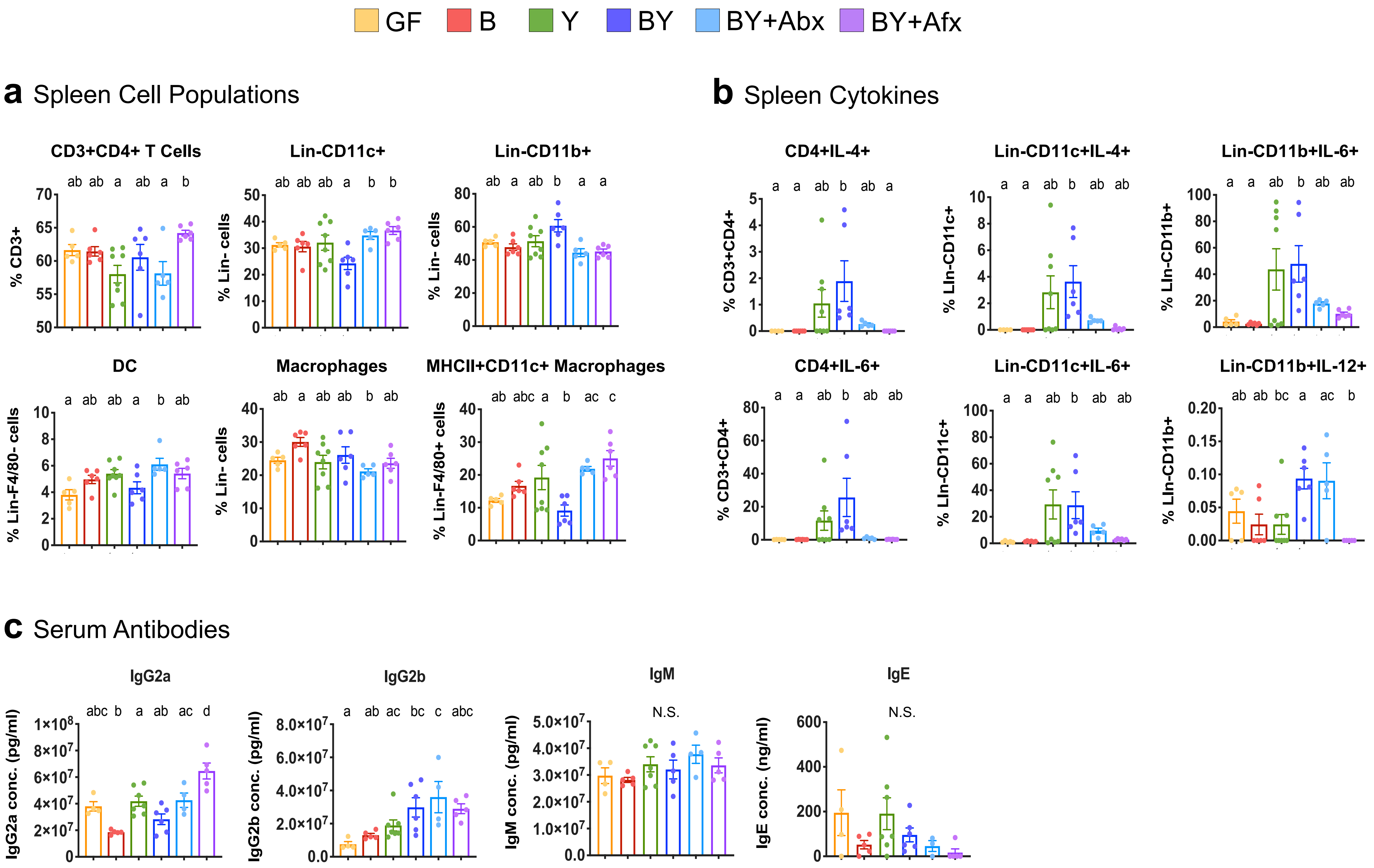

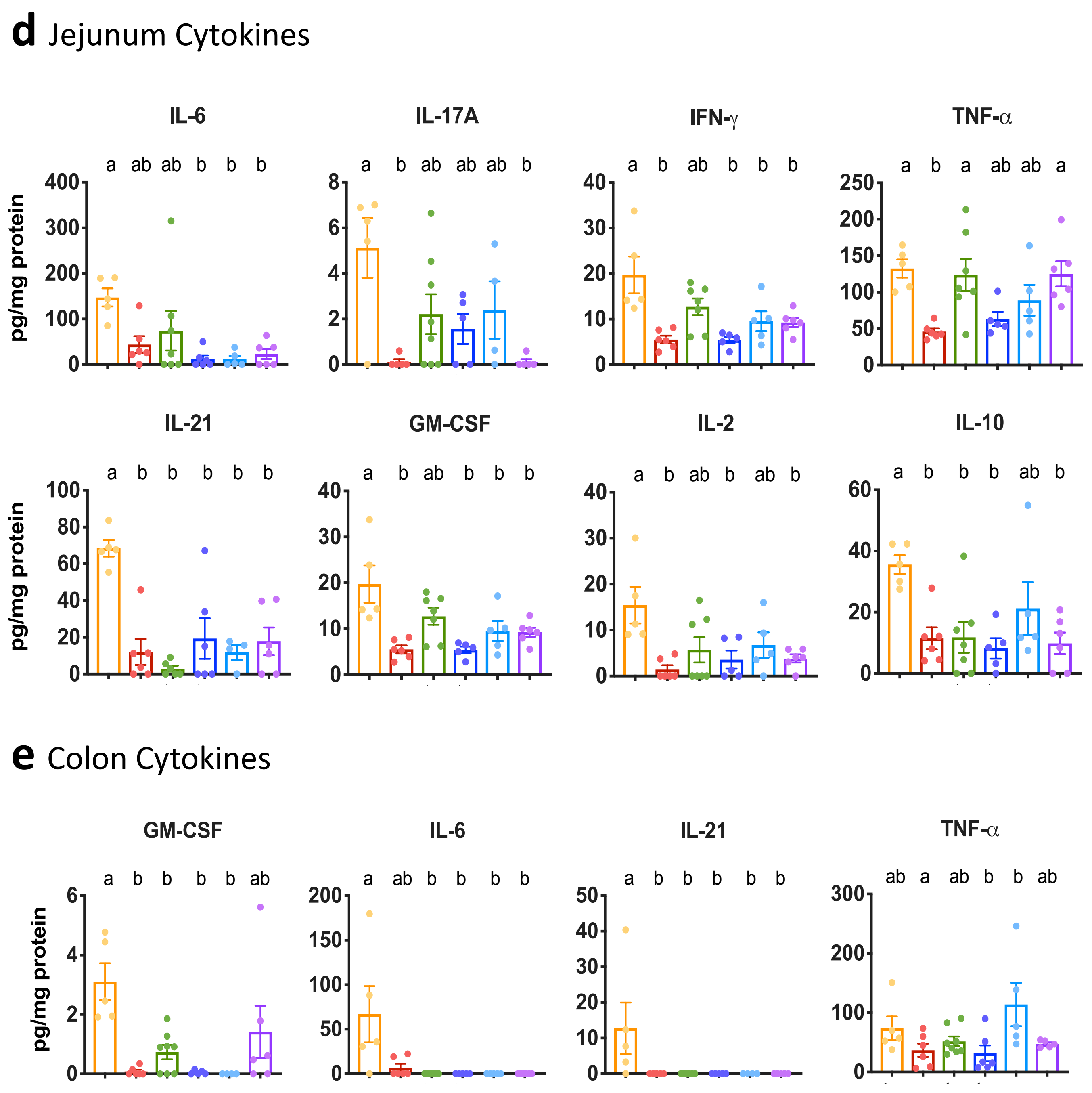

Supplementary Fig. 6: Fungal colonization impacts systemic and intestinal immunity. Percentage of **(a)** spleen cell populations and **(b)** cytokine-producing splenocytes from 4-week-old gnotobiotic mice. **(c)** Serum antibody concentrations detected by electrochemiluminescence (MSD – IgG2a, IgG2b and IgM) or ELISA (IgE). Cytokine concentration in **(d)** jejunum and **(e)** colon lysates detected by electrochemiluminescence (MSD). (a-e) Data expressed as mean ± S.E.M.; color denotes colonization treatment (GF = yellow, B = red, Y = green, BY = royal blue, BY+Abx = cyan blue, BY+Afx = purple); N_{GF}=5, N_B=6, N_Y=8, N_{BY}=6 , N_{Abx}=5, N_{Afx}=6; different letters above bars indicate statistically significant differences defined by one-way ANOVA and Tukey posthoc tests; $P<0.05$. Source data are provided as a Source Data file.

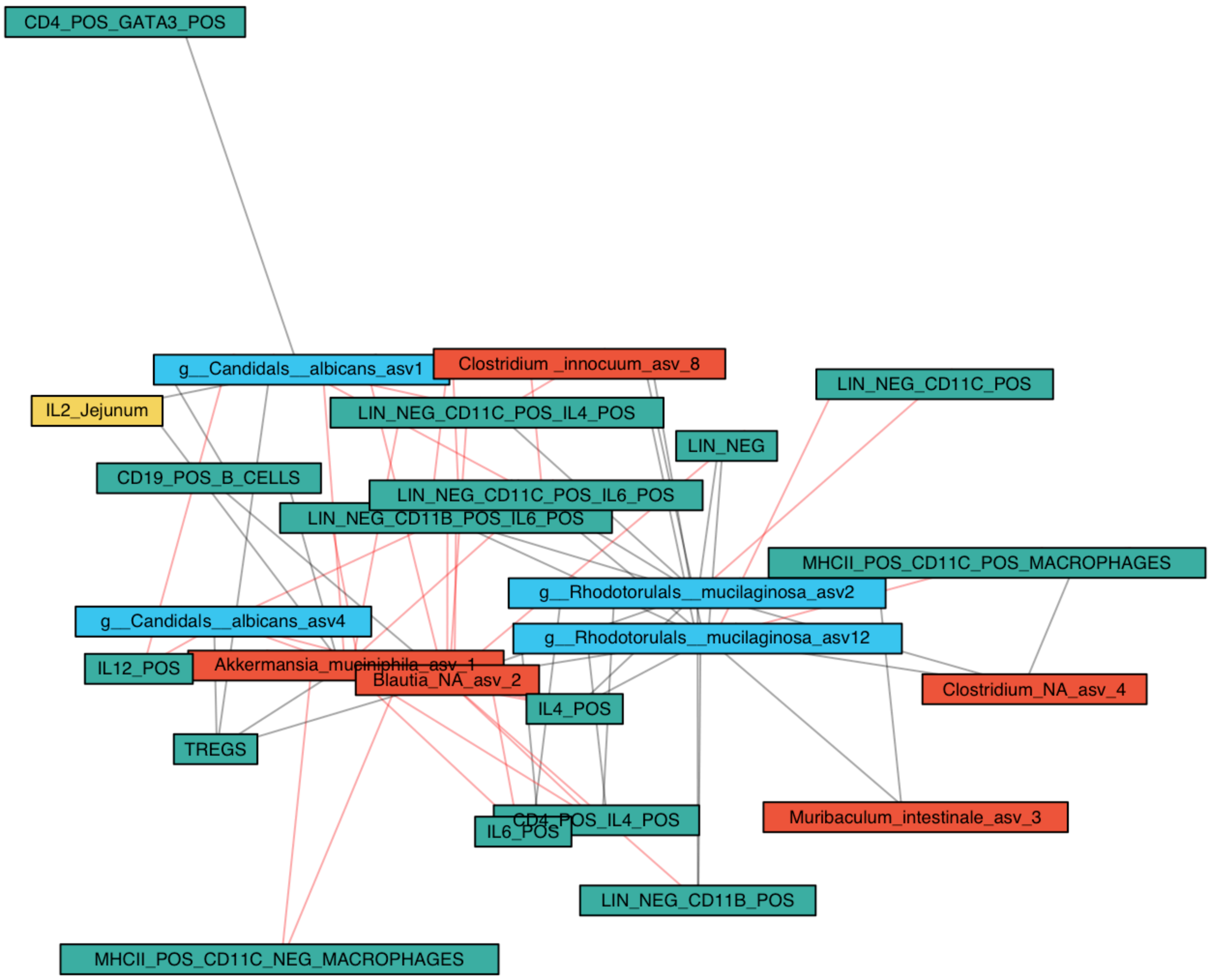

Supplementary Fig. 7: Network plot for microbial ASVs and immune features. Relevance network plots for bacteria (Persian red) and fungi (blue) ASVs with the systemic (green) and intestinal (yellow) immune features. Relevant correlations are denoted with a line (red=positive, black=negative) and defined by pair-wise similarity matrix for SGCCA.

Supplementary Table 1: Microbial species and primers used in this study.

**Oligo-MM$_{12}$ Consortium** [1]		**Source**
‘*Acutalibacter muris’* – KB18		Dr. K.D. McCoy
Akkermansia muciniphila – YL44		Dr. K.D. McCoy
‘*Bacteroides caecimuris*’ – I48		Dr. K.D. McCoy
Bifidobacterium longum subsp. *animalis* – YL2		Dr. K.D. McCoy
Blautia coccoides – YL58		Dr. K.D. McCoy
Clostridium clostridioforme – YL32		Dr. K.D. McCoy
Clostridium innocuum – I46		Dr. K.D. McCoy
Enterococcus faecalis – KB1		Dr. K.D. McCoy
Flavonifractor plautii – YL31		Dr. K.D. McCoy
Lactobacillus reuteri – I49		Dr. K.D. McCoy
Muribaculum intestinale – YL27		Dr. K.D. McCoy
‘*Turicimonas muris*’ – YL45		Dr. K.D. McCoy
**Fungal Strains**	**Fungal Guild** [2]	
Candida albicans	Undefined Saprotroph	Dr. D.R. Pillai/APL
Candida glabrata	Undefined Saprotroph	Dr. D.R. Pillai/APL
Candida parapsilosis	Undefined Saprotroph	Dr. D.R. Pillai/APL
Issatchenkia orientalis	Undefined Saprotroph	DSMZ (DSM 28721)
Rhodotorula mucilaginosa	Animal Endosymbiont-Animal Pathogen-Endophyte-Plant Pathogen-Undefined Saprotroph	DSMZ (DSM 70825)
**Universal 16S Primers** [3]		
U16Sfr: 5’-TCC TAC GGG AGG CAG CAG T-3’		
U16Srv: 5’- GGA CTA CCA GGG TAT CTA ATC CTG TT-3’		
**Fungal 18S Primers** [4]		
FR1: 5′-AIC CAT TCA ATC GGT AIT-3′		
FF390: 5′-CGA TAA CGA ACG AGA CCT-3′		
***C. albicans*-Specific Primers** [5]		
CALB1: 5'-TTT ATC AAC TTG TCA CAC CAG A-3'		
CALB2: 5'-ATC CCG CCT TAC CAC TAC CG-3'		
***R. mucilaginosa*-Specific Primers** [6]		
RM-5fw: 5′-GCG CTT TGT GAT ACA TTT TC-3′		
RM-3bw: 5′-CCA TTA TCC ATC CCG GAA AA-3′		

Abbreviations:

APL = Alberta Public Laboratories; DSMZ = Leibniz Institute DSMZ-German Collection of Microorganisms and Cell Cultures GmbH.

Supplementary Table 2: Permutational multivariate analysis of mice gut bacterial (16S) taxonomic community structure. Permutational multivariate analysis of variance stabilizing transformed community matrix testing the influence of cage ID, treatment type, and collection week. P-values of statistical significance defined by PERMANOVA on Bray-Curtis distances.

**Whole Dataset**		**16S Permutation**		
**Variable**	DF	F.Model	R_2 (%)	P-Value
**Treatment**	3	3.2603	15.14	<0.001
**Collection week**	1	12.085	14.08	<0.001
**Week 4**				
**Variable**	DF	F.Model	R_2 (%)	P-Value
**Treatment**	3	11.5081	62.91	<0.001
**Cage ID**	1	2.3526	4.28	0.083
**Week 9**				
**Variable**	DF	F.Model	R_2 (%)	P-Value
**Treatment**	3	3.1214	18.08	0.003
**Cage ID**	7	2.0595	27.84	0.005

Supplementary Table 3: Permutational multivariate analysis of mice gut fungal (ITS2) taxonomic community structure. Permutational multivariate analysis of variance stabilizing transformed community matrix testing the influence of cage ID, treatment type and collection week. P-values of statistical significance defined by PERMANOVA on Bray-Curtis distances.

**Whole Dataset**		**ITS2 Permutation**		
**Variable**	DF	F.Model	R_2 (%)	P-Value
**Treatment**	3	14.308	38.97	<0.001
**Collection week**	1	4.721	3.28	0.003
**Week 4**				
**Variable**	DF	F.Model	R_2 (%)	P-Value
**Treatment**	3	21.7798	74.25	<0.001
**Cage ID**	2	1.8284	4.15	0.132
**Week 9**				
**Variable**	DF	F.Model	R_2 (%)	P-Value
**Treatment**	3	56.206	65.85	<0.001
**Cage ID**	7	8.349	22.82	<0.001

Supplementary Table 4: Metabolites with statistically significant differences detected among gnotobiotic groups. List of 99 metabolites with statistically significant differences in relative abundance among gnotobiotic groups. Metabolites in fecal samples were extracted in 50% methanol solution and quantified by LC-MS in a Q Exactive HF Hybrid Quadrupole-Orbitrap Mass Spectrometer coupled to a Vanquish UHPLC System (see methods). Statistically significant differences determined by ANOVA with Fisher's post-hoc FDR correction; $P<0.05$.

Compound	F-Value	P-Value	-Log10(P)	FDR
SHIKIMATE	712.72	3.25E-30	29.489	4.38E-28
L-HISTIDINE	127.89	2.73E-19	18.564	1.79E-17
L-ASPARAGINE	124.56	3.97E-19	18.401	1.79E-17
SUCROSE	111.99	1.80E-18	17.745	6.07E-17
5-HYDROXY-L-TRYPTOPHAN	97.022	1.36E-17	16.866	3.68E-16
3-SULFINO-L-ALANINE	94.462	1.98E-17	16.703	4.46E-16
6-HYDROXYNICOTINATE	90.622	3.54E-17	16.450	6.84E-16
THYMIDINE	87.529	5.76E-17	16.240	9.71E-16
N-FORMYL-L-METHIONINE	86.743	6.53E-17	16.185	9.79E-16
NICOTINATE	84.825	8.91E-17	16.050	1.20E-15
GUANOSINE	80.509	1.84E-16	15.735	2.26E-15
N-ACETYL-L-PHENYLALANINE	73.039	7.06E-16	15.151	7.95E-15
N-ACETYL-L-ALANINE	71.962	8.66E-16	15.062	8.46E-15
L-CARNOSINE	71.895	8.78E-16	15.057	8.46E-15
STACHYOSE	62.985	5.35E-15	14.272	4.70E-14
5-OXO-D-PROLINE	62.797	5.57E-15	14.254	4.70E-14
DEOXYCYTIDINE	62.437	6.02E-15	14.220	4.78E-14
3-METHYL-2-OXOVALERIC ACID	58.466	1.46E-14	13.835	1.10E-13
D-GLUCOSAMINE-6-PHOSPHATE	54.791	3.50E-14	13.456	2.49E-13
URATE	51.999	7.03E-14	13.153	4.55E-13
4-METHYL-2-OXO-PENTANOIC ACID	51.969	7.08E-14	13.150	4.55E-13
D-RAFFINOSE	51.632	7.73E-14	13.112	4.74E-13
L-ASPARTATE	50.066	1.16E-13	12.935	6.82E-13
ADENINE	48.540	1.75E-13	12.757	9.85E-13
L-GLUTAMINE	41.542	1.34E-12	11.873	7.23E-12
MANNITOL	39.503	2.56E-12	11.592	1.33E-11
L-SERINE	36.829	6.27E-12	11.203	3.13E-11
L-LYSINE	36.149	7.94E-12	11.100	3.83E-11
4-HYDROXYBENZOATE	35.757	9.11E-12	11.040	4.19E-11
N-ACETYL-DL-GLUTAMIC ACID	35.698	9.30E-12	11.031	4.19E-11
THYMINE	34.790	1.29E-11	10.890	5.61E-11
N-ALPHA-ACETYL-L-ASPARAGINE	32.512	3.01E-11	10.522	1.27E-10
ITACONATE	31.191	5.03E-11	10.299	2.06E-10
L-ORNITHINE	28.390	1.59E-10	9.7981	6.32E-10
L-ARGININE	28.185	1.74E-10	9.7599	6.71E-10
XANTHOSINE	28.062	1.83E-10	9.7369	6.87E-10
TAURINE	27.225	2.64E-10	9.5781	9.64E-10
4-HYDROXY-2-QUINOLINECARBOXYLIC ACID	25.413	6.02E-10	9.2204	2.14E-09

Compound (Suppl. Table 4 continued)	F-Value	P-Value	-Log10(P)	FDR
SUCCINATE	24.363	9.91E-10	9.0038	3.43E-09
L-ARABITOL	22.336	2.73E-09	8.5644	9.20E-09
5-DEOXYADENOSINE	21.599	4.01E-09	8.3971	1.32E-08
N-ACETYL-GLUTAMINE	19.308	1.42E-08	7.8478	4.56E-08
L-CYSTEINE	18.512	2.26E-08	7.6456	7.10E-08
CYTIDINE	17.608	3.91E-08	7.4084	1.20E-07
URACIL	16.126	1.00E-07	6.9999	3.00E-07
BIOTIN	15.928	1.14E-07	6.9432	3.34E-07
URIDINE	15.145	1.93E-07	6.7146	5.54E-07
DEOXYURIDINE	14.147	3.88E-07	6.4114	1.09E-06
INOSINE	14.122	3.95E-07	6.4037	1.09E-06
NALPHA-ACETYL-L-LYSINE	13.628	5.65E-07	6.2482	1.52E-06
2-DEOXYADENOSINE	12.995	9.05E-07	6.0436	2.39E-06
N-ACETYL-L-LEUCINE	12.766	1.08E-06	5.9676	2.77E-06
S-CARBOXYMETHYL-L-CYSTEINE	12.755	1.09E-06	5.9642	2.77E-06
XANTHINE	11.142	3.94E-06	5.4050	9.84E-06
GLUCOSAMINATE	10.722	5.61E-06	5.2514	1.38E-05
L-CYSTATHIONINE	10.501	6.77E-06	5.1693	1.63E-05
N-ACETYL-D-TRYPTOPHAN	9.8940	1.15E-05	4.9381	2.73E-05
L-THREONINE	9.4238	1.76E-05	4.7534	4.11E-05
N-ACETYL-L-THREONINE	8.8196	3.10E-05	4.5085	7.07E-05
N-METHYL-D-ASPARTIC ACID	8.8062	3.14E-05	4.5029	7.07E-05
PTERIN	7.7336	9.02E-05	4.0449	0.0001996
D-GLUCURONIC ACID	7.5490	0.0001089	3.9630	0.0002371
SN-GLYCEROL-3-PHOSPHATE	7.1176	0.0001708	3.7675	0.0003660
FUMARATE	7.0042	0.0001926	3.7152	0.0004064
CREATINE	6.9724	0.0001993	3.7005	0.0004139
4-IMIDAZOLEACETIC ACID	6.9282	0.00020895	3.6800	0.0004274
L-ALANINE	6.8943	0.00021668	3.6642	0.0004366
D-GLUCOSE-6-PHOSPHATE	6.8636	0.00022397	3.6498	0.0004446
N-ACETYL-DL-SERINE	6.7970	0.00024062	3.6187	0.0004708
DOCOSAHEXAENOIC ACID	6.5938	0.00030014	3.5227	0.0005729
GUANINE	6.5903	0.00030131	3.5210	0.0005729
DL-5-HYDROXYLYSINE	6.5541	0.00031351	3.5038	0.0005878
XANTHURENIC ACID	6.4717	0.00034331	3.4643	0.0006349
L-TYROSINE	6.2399	0.00044433	3.3523	0.0008010
MALEIC ACID	6.2386	0.00044497	3.3517	0.0008010
3-METHYLADENINE	5.9952	0.00058598	3.2321	0.0010409
OPHTHALMIC ACID	5.8025	0.00073105	3.1361	0.0012817
3-UREIDOPROPIONATE	5.6646	0.00085799	3.0665	0.0014848
THYMIDINE-5-MONOPHOSPHATE	5.6538	0.00086889	3.0610	0.0014848
L-CYSTEIC ACID	5.5149	0.0010226	2.9903	0.0017069
L-LEUCINE	5.5137	0.0010241	2.9896	0.0017069
SUBERIC ACID	5.4937	0.0010485	2.9794	0.0017262
L-GLUTAMIC ACID	5.4328	0.0011268	2.9481	0.0018328
2-METHYLMALEATE	4.9243	0.0020812	2.6817	0.0033447

Compound (Suppl. Table 4 continued)	F-Value	P-Value	-Log10(P)	FDR
L-PHENYLALANINE	4.7888	0.0024600	2.6091	0.0039071
HYPOXANTHINE	4.7512	0.0025775	2.5888	0.0040276
DEHYDROASCORBATE	4.7456	0.0025956	2.5858	0.0040276
D-PANTOTHENIC ACID	4.6444	0.0029448	2.5309	0.0045152
N-ACETYL-DL-METHIONINE	4.6358	0.0029767	2.5263	0.0045152
ALPHA-D-GLUCOSE-1-PHOSPHATE	4.5955	0.0031311	2.5043	0.0046966
L-METHIONINE	4.3213	0.0044328	2.3533	0.0065761
3-HYDROXYBUTANOIC ACID	4.0014	0.0067064	2.1735	0.0098410
4-GUANIDINOBUTANOATE	3.6778	0.0102880	1.9877	0.0149340
FERULATE	3.5359	0.0124450	1.9050	0.0178740
INDOLE-3-ACETIC ACID	3.4546	0.0138920	1.8572	0.0197410
AZELAIC ACID	3.4000	0.0149600	1.8251	0.0210380
TRANS-CINNAMALDEHYDE	3.1908	0.0199190	1.7007	0.0277220
N-AMIDINO-L-ASPARTATE	3.0241	0.0250830	1.6006	0.0345530
L-TRYPTOPHAN	2.8945	0.0300480	1.5222	0.0409750

Supplementary Table 5: Metabolites with statistically significant differences detected between Y and GF gnotobiotic groups. List of 22 metabolites with statistically significant differences in relative abundance between Y and GF groups. Statistically significant differences determined by Two-Sided T-test with FDR correction; P<0.05.

Compound	T-Stat	P-Value	-Log10(P)	FDR
NICOTINIC ACID	-25.689	2.29E-07	6.6395	2.98E-05
3-HYDROXYBUTYRIC ACID	-8.1624	0.000182	3.7400	0.011828
FUMARIC ACID	-6.6632	0.000553	3.2575	0.023949
L-ASPARAGINE	5.8051	0.001146	2.9408	0.032220
KETOLEUCINE	5.6874	0.001275	2.8946	0.032220
INDOLEACETIC ACID	-5.5199	0.001487	2.8277	0.032220
ACETYLALANINE	5.3291	0.001780	2.7496	0.033052
THYMINE	-5.0012	0.002449	2.6109	0.037887
CARNOSINE	4.9327	0.002623	2.5812	0.037887
6-HYDROXYNICOTINIC ACID	4.7618	0.003121	2.5058	0.039944
SUCCINIC ACID	-4.6555	0.003484	2.4580	0.039944
L-GLUTAMIC ACID	4.5445	0.003915	2.4073	0.039944
URIDINE	4.4241	0.004451	2.3515	0.039944
N-ACETYL-D-TRYPTOPHAN	4.4194	0.004474	2.3493	0.039944
L-SERINE	4.3764	0.004686	2.3292	0.039944
UROCANIC ACID	4.3323	0.004916	2.3084	0.039944
DOCOSAHEXAENOIC ACID	4.1881	0.005761	2.2395	0.042696
SHIKIMIC ACID	4.1649	0.005912	2.2283	0.042696
SUBERIC ACID	4.0754	0.006536	2.1847	0.044720
CITRACONIC ACID	4.0233	0.006933	2.1591	0.045064
L-CYSTATHIONINE	-3.9504	0.007534	2.1230	0.046639
N-ACETYL-L-PHENYLALANINE	3.8837	0.008136	2.0896	0.048077

References (Supplementary Table 1):

1. Brugiroux, S. *et al.* Genome-guided design of a defined mouse microbiota that confers colonization resistance against *Salmonella enterica* serovar *Typhimurium*. *Nat Microbiol* **2**, 16215 (2016).
2. Nguyen, N.H. *et al*. FUNGuild: An open annotation tool for parsing fungal community datasets by ecological guild. *Fungal Ecol* **20**, 241-248 (2016).
3. Nadkarni, M.A., Martin, F.E., Jacques, N.A. & Hunter, N. Determination of bacterial load by real-time PCR using a broad-range (universal) probe and primers set. *Microbiology* **148**, 257-266 (2002).
4. Prevost-Boure, N.C. *et al.* Validation and application of a PCR primer set to quantify fungal communities in the soil environment by real-time quantitative PCR. *PLoS One* **6**, e24166 (2011).
5. Zhang J. *et al.* Development of *Candida*-specific real-time PCR assays for the detection and identification of eight medically important *Candida* species. *Microbiol Insights* **9**, 21-8.
6. Díaz C. *et al.* Characterization and dynamic behavior of wild yeast during spontaneous wine fermentation in steel tanks and amphorae. *Biomed Res Int* **2013**, 540465 (2013).
