# Supplementary material for: Intestinal fungi are causally implicated in microbiome assembly and immune development in mice
Source: Nat Commun. 2020 May 22;11:2577. doi: 10.1038/s41467-020-16431-1 (PMC7244730; doi:10.1038/s41467-020-16431-1)
Supplement: Supplementary file 3 — Reporting Summary [file 41467_2020_16431_MOESM3_ESM.pdf]

natureresearch

Corresponding author(s): Marie-Claire Arrieta

Last updated by author(s): Apr 24, 2020

Reporting Summary

Nature Research wishes to improve the reproducibility of the work that we publish. This form provides structure for consistency and transparency in reporting. For further information on Nature Research policies, see Authors & Referees and the Editorial Policy Checklist .

Statistics

For all statistical analyses, confirm that the following items are present in the figure legend, table legend, main text, or Methods section.

n/a	Confirmed	
☐	☒	The exact sample size (*n*) for each experimental group/condition, given as a discrete number and unit of measurement
☐	☒	A statement on whether measurements were taken from distinct samples or whether the same sample was measured repeatedly
☐	☒	The statistical test(s) used AND whether they are one- or two-sided<br>*Only common tests should be described solely by name; describe more complex techniques in the Methods section.*
☐	☒	A description of all covariates tested
☐	☒	A description of any assumptions or corrections, such as tests of normality and adjustment for multiple comparisons
☐	☒	A full description of the statistical parameters including central tendency (e.g. means) or other basic estimates (e.g. regression coefficient) AND variation (e.g. standard deviation) or associated estimates of uncertainty (e.g. confidence intervals)
☐	☒	For null hypothesis testing, the test statistic (e.g. *F*, *t*, *r*) with confidence intervals, effect sizes, degrees of freedom and *P* value noted<br>*Give P values as exact values whenever suitable.*
☒	☐	For Bayesian analysis, information on the choice of priors and Markov chain Monte Carlo settings
☒	☐	For hierarchical and complex designs, identification of the appropriate level for tests and full reporting of outcomes
☒	☐	Estimates of effect sizes (e.g. Cohen's *d*, Pearson's *r*), indicating how they were calculated

Our web collection on statistics for biologists contains articles on many of the points above.

Software and code

Policy information about availability of computer code

Data collection	The pooled and indexed amplicon libraries were denatured, diluted, and sequenced in paired-end modus on an Illumina MiSeq (Illumina Inc., San Diego, USA). 16S and ITS2 rRNA sequencing were performed at Microbiome Insights.
Data analysis	Sequences were checked for quality, trimmed, merged, and checked for chimeras using the DADA2 v1.10.1 pipelines for 16S or ITS2 and phyloseq v.1.26.1 as packages for R (R Development Core Team; http://www.R-project.org) in RStudio v.1.1463. We built bacterial and fungal community matrices from the resulting unique Amplicon Sequence Variants (ASV) based on the UNITE v.8.0 (fungi) and SILVA v.132 (bacteria) databases. Complete workflow tutorials can be found at https://benjjneb.github.io/dada2/tutorial.html and benjjneb.github.io/dada2/ITS_workflow.html. For community-level analyses (alpha- and beta-diversity and relative abundance), the most abundant 22 bacterial and 16 fungal ASVs, which explained >98% of all assigned reads, were merged by species name.<br><br>PCA, ANOVA, T-tests, and pathway analysis of fecal metabolites originating from different mice groups was performed by using Metaboanalyst v.4.0. For all other biological samples, graphs and statistical analyses were made using either RStudio v. 1.1.463 or Prism version 8.1.2.

For manuscripts utilizing custom algorithms or software that are central to the research but not yet described in published literature, software must be made available to editors/reviewers. We strongly encourage code deposition in a community repository (e.g. GitHub). See the Nature Research guidelines for submitting code & software for further information.

Data

Policy information about availability of data

All manuscripts must include a data availability statement. This statement should provide the following information, where applicable:

- Accession codes, unique identifiers, or web links for publicly available datasets
- A list of figures that have associated raw data
- A description of any restrictions on data availability

The source data underlying all presented Figures and Supplementary Figures and Tables are provided as a Source Data file. 16S and ITS2 sequence reads and supporting metadata were deposited to European Nucleotide Archive (ENA), https://www.ebi.ac.uk/ena/browser/view/PRJEB35163 (study accession numbers PRJEB35163/ERP118175). Metabolomics mass spectral raw data were deposited to MetaboLights, https://www.ebi.ac.uk/metabolights/MTBLS1679 (study identifier MTBLS1679). All microbial strains used are kept as stocks in the Arrieta lab and can be shared when requested.

Field-specific reporting

Please select the one below that is the best fit for your research. If you are not sure, read the appropriate sections before making your selection.

[x] Life sciences [ ] Behavioural & social sciences [ ] Ecological, evolutionary & environmental sciences

For a reference copy of the document with all sections, see nature.com/documents/nr-reporting-summary-flat.pdf

Life sciences study design

All studies must disclose on these points even when the disclosure is negative.

Sample size

Sample size was determined from previous experience and published data on required number of mice per group for colitis (PMIDs: 30850233 and 27237365) and lung inflammation (PMIDs: 26424567, 24439901, 21471101, and 15618138) models. All mice birthed from breeding pairs were used in the experiments.

Data exclusions

One female mice was removed from all barrier assessment and intestinal physiology analyses as demonstrating signs of disease (specific values excluded highlighted in Source Data file provided). This specific female had quite reduced body mass and showed signs of being Beyond this, all other animals were included in experiments but data outliers were identified and removed based on a 1% ROUT method in Prism version 8.1.2 (GraphPad Software, La Jolla, CA, USA).

Replication

qPCR experiments were performed in duplicate and data combined. Murine colonization, early life immunity assessment, and OVA-challenge model were repeated, for a total of two independent experiments. All attempts of replication were successful and provided similar results. DSS model, barrier assessment, and intestinal physiology experiments were not repeated and data presented are from one experiment.

Randomization

Individuals allocated in sample based on breeding rates and birth. Mice were separated in different isolators based on colonization group (i.e: B, Y, BY, GF).

Blinding

Investigators were blinded through several steps of data collection and analyses. An individual not involved in the analyses created an alpha-numeric code and tracking sheet before delivering the samples to the person running the experiment. This was performed for all barrier assessment, intestinal physiology, OVA response, DSS pathology scoring, and metabolomics data acquisition.

Other experiments were done in a semi-blinded way, such as sample preparation for sequencing, flow cytometry, immune assessment in MSD and ELISA runs, and qPCR. For these, samples were obtained by the same investigators that performed the experiment and/or analyzed the data. Sequencing and flow cytometry analyses were performed blinded and metadata for sample identification merged at the end. Cytokine and antibody assessment in MSD and ELISA and qPCR assays were performed in a non-blinded way, although they relied in standard curves that were applied to the whole experiment, ensuring data fidelity. The non-blinded experiments were performed by E.v.T.B and M.W.G., who were involved in most steps of sample preparation, plus had appropriate training or access to the equipments used for data acquisition.

Reporting for specific materials, systems and methods

We require information from authors about some types of materials, experimental systems and methods used in many studies. Here, indicate whether each material, system or method listed is relevant to your study. If you are not sure if a list item applies to your research, read the appropriate section before selecting a response.

Materials & experimental systems

n/a	Involved in the study	
[ ]	[x]	Antibodies
[x]	[ ]	Eukaryotic cell lines
[x]	[ ]	Palaeontology
[ ]	[x]	Animals and other organisms
[x]	[ ]	Human research participants
[x]	[ ]	Clinical data

Methods

n/a	Involved in the study	
[x]	[ ]	ChIP-seq
[ ]	[x]	Flow cytometry
[x]	[ ]	MRI-based neuroimaging

Antibodies

Antibodies used	The following antibodies were used: PE-Cy7 anti-mouse CD19 (clone 1D3; BD Biosciences), PerCP-Cy5.5 anti-CD11b (M1/70; BD Bioscience), APC-H7 anti-mouse CD8a (53-6.7; BD Bioscience), Alexa Fluor 700 anti-mouse CD3 (17A2; BD Bioscience), V500 anti-mouse CD4 (RM4-5; BD Biosciences), Brilliant Violet 421 anti-mouse CD25 (PC61; BD Biosciences), Brilliant Violet 421 anti-mouse CD11c (HL3; BD Biosciences), PE anti-Gata3 (L50-823; BD Biosciences), PE anti-mouse IL-17A (TC11-18H10; BD Biosciences), PE anti-mouse IL-12/23 (C15.6; BD Biosciences), PE anti-mouse F4/80 (T45-2342; BD Biosciences), Alexa Fluor 488 anti-mouse IL-4 (11B11; BD Biosciences), Alexa Fluor 488 anti-mouse FoxP3 (MF23; BD Biosciences), Alexa Fluor 488 anti-mouse INF-g (XmG1.2; BD Biosciences), Alexa Fluor 488 anti-mouse I-A/I-E (M5/114.15.2; BD Biosciences), eFluor 660 anti-mouse IL-13 (eBio13A; eBioscience, San Diego, CA), APC anti-mouse IL-10 (JES5-16E3; BD Biosciences), APC anti-mouse IL-6 (MP5-20F3; BD Biosciences), and APC anti-mouse CD103 (M290; BD Biosciences). All antibodies for surface and intracellular markers were diluted 200 times, with the exception of CD8a that was diluted 100 times.
Validation	The primary antibodies were tested for proper staining and compensation adjustment by different staining controls, although no specific titration was performed. Panel was designed with support from Technical Applications Specialist from BD Biosciences. Test run with suggested dilution for the antibodies (200 times dilution), including single-stained cell suspensions and fluorescence menus one (FMO) controls, was run in BD FACSCanto II (BD Biosciences), in the Nicole Perkins Microbial Communities Core Lab, Snyder Institute for Chronic Diseases, University of Calgary. The test run was sufficient to confirm proper staining and identify compensation issues. Other than CD8a that was concentrated 2x (100 times final dilution) for brightness adjustment, other antibodies demonstrated proper marking and were therefore used as described.

Animals and other organisms

Policy information about studies involving animals; ARRIVE guidelines recommended for reporting animal research

Laboratory animals	This study made use of C57BL/6J mice. Age is reported through the manuscript. Breeding pairs (M:F 2:1 ratio, age 5-12 weeks old) were purchased from the International Microbiome Centre. Other than intestinal physiology experiments that made use of solely females (dams), all other experiments had mixed male and female sexes (information included in the Source Data File). Following breeding, pups were euthanized at age 4 weeks (early like immune assessment), 7 weeks (DSS protocol), or 9-10 (OVA challenge).
Wild animals	This study did not involve wild animals.
Field-collected samples	This study did not involve samples collected from the field.
Ethics oversight	All experiments were conducted under protocols approved by the University of Calgary Animal Care Committee, following the guidelines of the Canadian Council on Animal Care.

Note that full information on the approval of the study protocol must also be provided in the manuscript.

Flow Cytometry

Plots

Confirm that:

- [x] The axis labels state the marker and fluorochrome used (e.g. CD4-FITC).
- [x] The axis scales are clearly visible. Include numbers along axes only for bottom left plot of group (a 'group' is an analysis of identical markers).
- [x] All plots are contour plots with outliers or pseudocolor plots.
- [x] A numerical value for number of cells or percentage (with statistics) is provided.

Methodology

Sample preparation	Spleens were surgically removed and processed for flow cytometry. Spleens were kept in ice-cold RPMI 1640 + GlutaMax-I medium (Thermo-Fisher) and were macerated in GentleMACS C tubes (Miltenyi Biotec, Bergisch Gladbach, Germany). Cellular macerate was filtered through a 100 µm cell strainer and red blood cells were lysed in 5 ml 1x RBC Lysis Buffer (BioLegend, San Diego, CA, USA). Splenocytes were resuspended in ice-cold cell growth media constituted of RPMI 1640 + GlutaMax-I medium (Thermo-Fisher) supplemented with 10% heat-inactivated Fecal Bovine Serum (Thermo-Fisher), 2000 U Penicillin-Streptomycin (Thermo-Fisher), and 50 µM 2-Mercaptoethanol (Thermo-Fisher). Isolated splenocytes were stained with Fixable Viability Stain (FVS575V; BD Biosciences) and array of intra and extracellular markers for immune cell characterization.
Instrument	Samples were run in BD FACSCanto II (BD Biosciences), in the Nicole Perkins Microbial Communities Core Lab, Snyder Institute for Chronic Diseases, University of Calgary.
Software	Analysis performed in FlowJoTM version 10.5.3.
Cell population abundance	Abundance of relevant populations and parental gates are specified in each figure. Data obtained from total splenocytes in samples following scattering selection and exclusion of viability stain positive population.

Gating strategy

SSC/FSC (off all events) > Viability/FSC (of lymphocyte population) > CD3/CD19 (of FVS negative population) > CD4/CD8 (of CD3 positive population) > CD25/FoxP3 (of CD3 positive CD4 positive population) > CD11b/CD11c (of CD3 negative CD19 negative population) > F480/CD11c (of CD3 negative CD19 negative population) > IAIE/CD11c (of CD3 negative CD19 negative F480 positive CD11c positive population) > IAIE/CD11c (of CD3 negative CD19 negative F480 negative population) > CD103/CD11b (of of CD3 negative CD19 negative F480 negative IA/IE positive CD11c positive population)

☒ Tick this box to confirm that a figure exemplifying the gating strategy is provided in the Supplementary Information.
